# A novel proximal 3q29 chromosome microdeletion in a Chinese patient with Chiari malformation type II and Sprengel’s deformity

**DOI:** 10.1186/s13039-018-0358-4

**Published:** 2018-01-24

**Authors:** Shuai Guo, Xue-Feng Fan, Jie-Yuan Jin, Liang-Liang Fan, Lei Zeng, Zheng-Bing Zhou, Rong Xiang, Ju-Yu Tang

**Affiliations:** 1 Department of orthopaedics, Xiangya Hospital of Central South University, Changsha, 410078 People’s Republic of China; 2 School of Life Sciences, Central South University, Changsha, 410013 People’s Republic of China

**Keywords:** Chiari malformation, Sprengel’s deformity, XXYLT1, ACAP2, 3q29 microdeletion

## Abstract

**Background:**

Chiari malformation type II (CM-II) is mainly characterized by elongation and descent of the cerebellum through the foramen magnum into the spinal canal. Moreover, CM-II is uniquely associated with myelomeningocele. Sprengel’s deformity refers to the malposition of the scapula, i.e. scapular elevation which is sometimes accompanied with scapula dysplasia. Although few familial cases of CM-II and Sprengel’s deformity have been previously reported, both of these defects are considered to be sporadic, thus the exact etiology and causative genes have largely remained unknown.

**Case presentation:**

The patient was diagnosed with CM-II accompanied with Sprengel’s deformity. Further genetic investigation revealed a novel 666 kb microdeletion located in 3q29 (chr3:194,532,035–195,198,585; Hg19). Subsequently, genes within the affected region were summarized, and *XXYLT1* and *ACAP2* were identified as the candidate genes.

**Conclusion:**

We reported a case of a patient with CM-II and Sprengel’s deformity harboring a microdeletion in 3q29. This case highlights the importance of 3q29 in early neural and skeletal development, as well as expands the phenotype spectrum of this rare disorder.

## Background

Chiari malformation type II (CM-II; OMIM #207950) is a rare congenital defect, characterized by elongation and descent of the cerebellum through the foramen magnum into the spinal canal. Other cerebral structures including pons, medulla, and fourth ventricle can also be affected, leading to the brain stem compression, or obliteration of the fourth ventricle and cisterna magna [1, 2]. Moreover, CM-II is uniquely associated with myelomeningocele (OMIM #182940), another congenital malformation identified as unfused neural tube occurring between postovulatory days 21 and 27 [3]. CM-II associated myelomeningocele is mainly observed in thoracic segments, while the nonsyndromic myelomeningocele often affects lumbosacral portions [2, 4, 5]. Sprengel’s deformity (OMIM #184400) is the most common congenital abnormality of the shoulder girdle [6]. It refers to the congenital elevation of the scapula, sometimes accompanied with scapula dysplasia. What’s more, complications like regional muscle hypoplasia or atrophy can occur, leading to limited shoulder movement. Although several familial cases of CM-II or Sprengel’s deformity have been reported, both defects are widely considered to be sporadic, due to the unknown etiology and undetermined causal genes [7–10].

Matsuoka et al. have studied the origin of neck and shoulder in mice model [11]. Their results indicated that the parts of the skeleton affected in CM-I/II, Sprengel’s deformity and the Klippel­Feil syndrome all derive from a specific population of neural crest cells, suggesting these diseases might have a similar developmental origin. However, research on the association between spinal dysraphism and Sprengel’s deformity is scarce [12].

Herein, we reported a case of a patient diagnosed with CM-II accompanied with Sprengel’s deformity. One-copy deletion of 3q29 region (chr3: 194,532,035–195,198,585; Hg19) was identified. The affected region contains two genes, *XXYLT1* (xyloside xylosyltransferase 1, also known as *C3orf21*; OMIM #614552) and *ACAP2* (Arf-gap with coiled-coil, ankyrin repeat, and pleckstrin homology domains-2, also known as *CENTB2*; OMIM #607766). *XXYLT1* encodes enzyme xyloside xylosyltransferase, which is responsible for the addition of xylose to O-glucosylated Notch epidermal growth factor (EGF)-like repeats, and mainly functions in Notch signaling transduction [13]. ACAP2 is the GTPase-activating proteins of Arf6 (ADP-ribosylation factor 6). ACAP2 mediates cellular events including endocytic recycling, phagocytosis, cytokinesis and neurite outgrowth through regulating Arf6, which is involved in the endosomal membrane trafficking and the actin cytoskeleton in the cell periphery [14].

## Case presentation

The Review Board of the Xiangya Hospital of the Central South University approved this study. The informed consent was obtained from each subject.

### Surgical Repairation

**Fig. 1:**
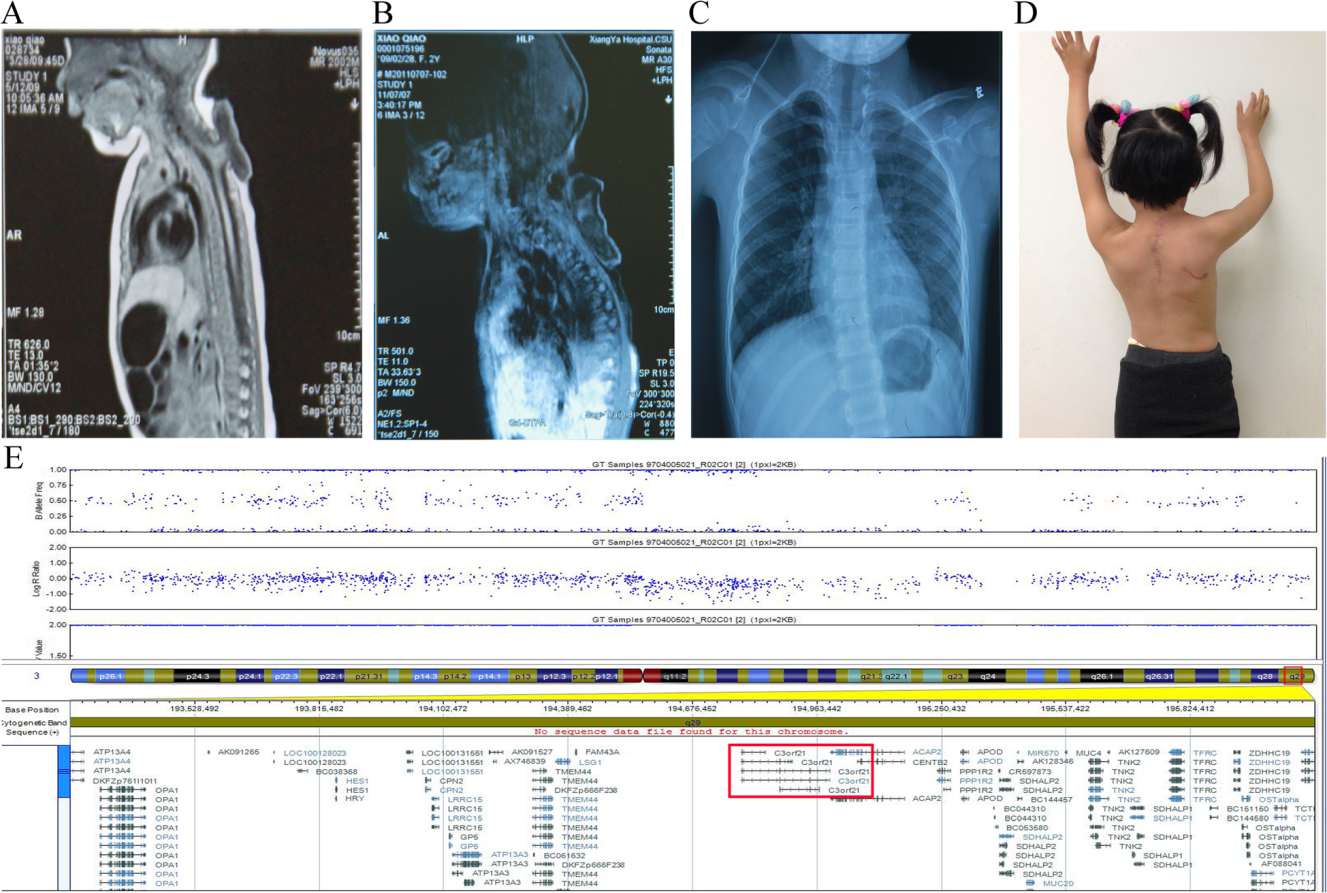
Phenotypic appearance of the affected individual in our study. **a** X-ray shows spinal meningocele in the cervicothoracic region in our patient at birth. **b** This X-ray was taken in 2011 when the proband came to our institution for spina bifida repair. It shows that besides myelomeningocele, the proband also had elongated and descended cerebellum. These results led to her diagnosis of Chiari malformation. **c** Patient’s X-ray from 2016, revealing an elevated right scapula. **d** After partial scapulectomy and collum costatectomy, the shoulder function was partially restored. **e** Human 660w–Quad SNP-array results. A 666 Kb microdeletion (chr3:194,532,035–195,198,585; Hg19) located in the 3q29 region. *XXYLT1* (*C3orf21*) is affected by this deletion

The patient, a 7-year-old girl from the Central-south region of China (Xiangtan, Hunan Province), was born to a non-consanguineous family. No family history of congenital defects was noted. The gestation process was uncomplicated by maternal illness or teratogen exposure. Our patient was born at 41 weeks by spontaneous vaginal delivery. The birth weight and length of the proband were normal, i.e. 2900 g (25th centile) and 43 cm (25th centile) respectively. Moreover, her head circumference was 34 cm (25th centile). Nonetheless, she was observed having spinal meningocele in the cervicothoracic region (Fig. 1a). No facial phenotypes were noted.

Her spinal defect was repaired at the age of two, i.e. in 2011 when she came to our institution. MRI and X-ray were performed as part of a comprehensive preoperative physical examination. MRI results showed an extended cerebellum, and brain stem extending into the foramen magnum (Fig. 1b). Based on her medical history of spina bifida, she was diagnosed with CM-II [15]. What’s more, X-ray showed soft tissue mass shadows in the cervicothoracic region, while the cardiac and pulmonary features were normal. In 2016, the patient came again to our institution due to left acromioclavicular dislocation and significantly elevated right scapula (Fig. 1c). Indentations were observed in the axilla and shoulder region, and were accompanied with muscular hypertonia. The physiological functions of her articulatio humeri were assessed, and the obtained results showed impaired pronation and supination. Subsequently, partial scapulectomy and collum costatectomy were performed. The procedure was smooth, and the patient partially regained shoulder function (Fig. 1d).

### Cytogenetic analysis

Five milliliters of peripheral blood were collected from the patient and her parents. The blood samples were subjected to lymphocyte culture as previously described [16]. Chromosome analysis was performed by conventional G-Banded techniques at the level of 550 bands. Karyotype analysis of the patient was normal. We hypothesized that CNVs might contribute to the multiple birth defects. Then, the genomic DNA was prepared using a DNeasy Blood & Tissue Kit (Qiagen, Valencia, CA) on the QIAcube automated DNA extraction robot (Qiagen, Hiden, Germany) [17]. Single nucleotide polymorphism (SNP)-array analysis was employed to test it (Human660W–Quad Chip, Bead station Scanner, and Genome Studio V2011 software) [18]. The call rates of the samples were greater than 99.5%. We performed a family-based copy number variations (CNVs) validation with the PennCNV algorithm to obtain authentic CNVs [19]. We identified a 666 kb microdeletion located in 3q29 (chr3:194,532,035–195,198,585; Hg19). This deletion might lead to haploinsufficiency of *XXYLT1* and *ACAP2*, due to function loss of the other allele (Fig. 1e). The function of these two genes is briefly summarized in the Table-1.

In conclusion, our patient was diagnosed with CM-II accompanied with Sprengel’s deformity. Surgical intervention was effective, with good postoperative recovery.

## Discussion

Herein, we described the clinical features and genotype of a patient with CM-II and Sprengel’s deformity. She was born with open spina bifida, and she received repair surgery at the age of two. The diagnosis of CM-II malformation was made with the use of magnetic resonance imaging (MRI). Five years later, she returned to our institution because of Sprengel’s deformity diorthosis. To determine the underlying genetic cause for our patient’s conditions, we performed SNP array and identified a novel 666 Kb microdeletion in the terminal 3q29 region (chr3: 194,532,035–195,198,585; Hg19).

Thus far, more than 40 cases of 3q29 microdeletion have been reported [20, 21]. Most patients share a common 2 Mb deletion from 196 Mb to 198 Mb, which are mainly characterized by psychiatric manifestations, including autism and schizophrenia. Three genes, *FBXO45* (OMIM #609112), *DLG1* (OMIM #601014), and *PAK2* (OMIM #605022), have been proposed as a cause of these mental disorders due to their roles in synaptic transmission [21, 22]. Other genes located in the affected region may also contribute to the pathogenic process.

**Fig. 2:**
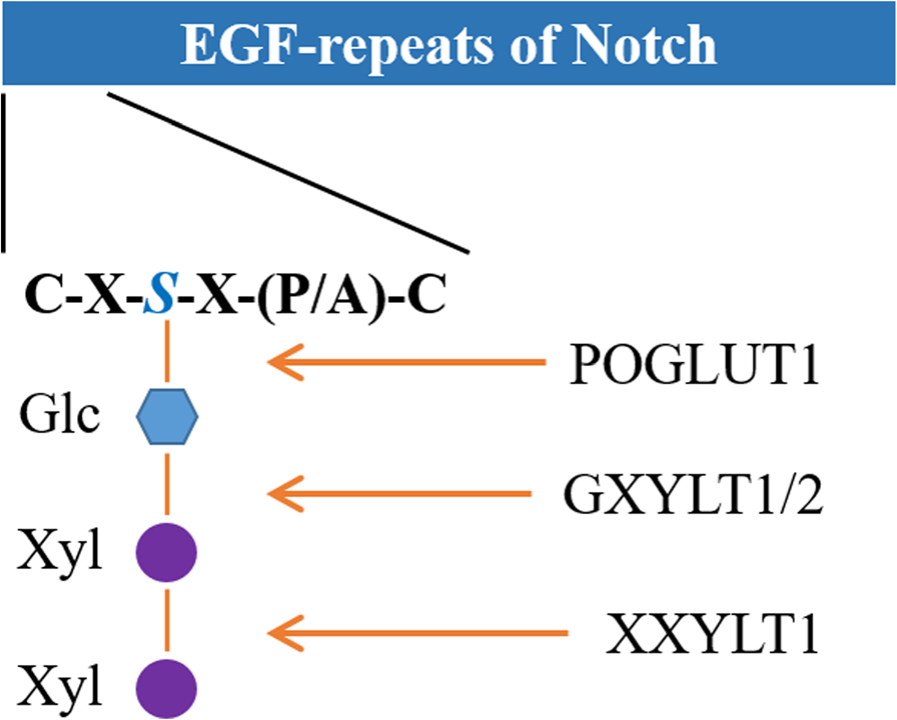
The O-glucose residues and the first xylose residues are added to EGF repeats with C-X-S-X-(P/A)-C motif of the Notch by POGLUT-1 and GXYLT1/2, respectively. The second xylosylation is mediated by XXYLT1

**Fig. 3:**
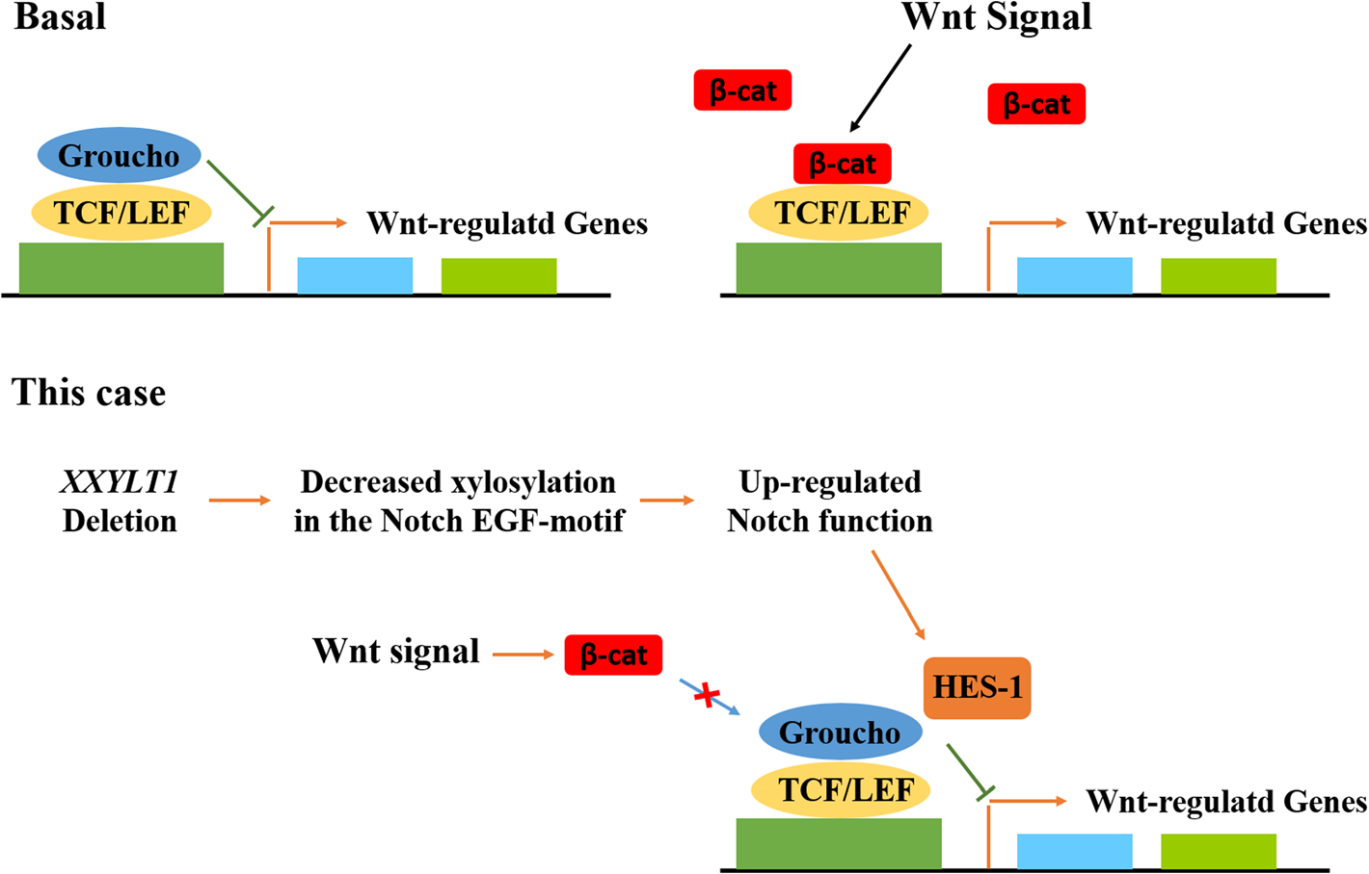
In the absence of β-catenin, TCF/LEF-1 and Groucho can form a complex and suppress transcriptional events. When Wnt signaling is activated, and β-catenin is stabilized, Groucho is displaced, and it binds TCF/LEF-1 causing a transcriptional activation. In our case, XXYLT1 deletion leads to impaired Notch xylosylation, causing abnormally up-regulated Notch function. This process decreases β-catenin and increases HES-1, which interacts with Groucho and prevents the following activation of gene transcriptions. As a result, osteoblastogenesis mediated by Wnt/β-catenin is inhibited by over-functional Notch signaling, which may contribute to the pathogenic process and finally lead to the congenital skeletal defects observed in our patient

However, in the present study, an atypical 3q29 deletion with the affected region range from 194 Mb to 195 Mb comprising two genes, *ACAP2* and *XXYLT1*, was detected (Table 1). *XXYLT1* encodes the protein Xyloside Xylosyltransferase, which is localized in the endoplasmic reticulum. This enzyme mediates xylosylation, a conserved post-translational modification characterized as the addition of the second xylose residue to EGF-like repeats with C-X-S-X-(P/A)-C motif of the Notch protein. The O-glucose residues and the first xylose residues are added by Protein O-glucosyltransferase 1 (POGLUT-1) and Glucoside Xylosyltransferase-1 or − 2 (GXYLT1/2) respectively (Fig. 2) [13]. Both glucosylation and xylosylation function as modulators of Notch signaling pathway. Previous studies in *Drosophila* and *Mus musculus* revealed that xylose residues on EGF16–20 can negatively regulate the surface expression of the Notch receptor, while overexpressed Notch1 can inhibit osteoblastogenesis by suppressing Wnt/β-Catenin signaling [13, 23, 24]. It is very likely that this 3q29-deletion induced *XXYLT1* dysfunction may impair EGF xylosylation, lead to up-regulated Notch signaling, and eventually result in impaired osteoblastogenesis, including spinal defects and Sprengel’s deformity (Fig. 3).

**Table 1:** Genes in the Identified 3q29 Deleted Region

Gene	Function
*ACAP2*	ACAP2 is an Arf-6 GTPase-activating protein. The main function of ACAP2 is to controlling the return of Arf-6 to the inactive GDP-bound state, which is very critical in Arf-6 function regulation.
*XXYLT1*	*XXYLT1* encodes an α-1,3-xylosyltransferase. It can catalyze the addition of the second xylose to elongate the xylose-glucose disaccharide in the extracellular domain of Notch proteins.

While *XXYLT1* mainly functions in Notch signaling during development, the other affected gene, *ACAP2*, is thought to participate in various cellular events including endocytic recycling, phagocytosis, cytokinesis and neurite outgrowth [14, 25]. ACAP2 is one of the Arf6-GAPs, and mainly functions as the inhibitor of Arf6 by catalyzing Arf6 into the inactive GDP-bound state. Previous study has revealed that ACAP2 regulates neurite outgrowth by inactivating Arf6 at the pericentrosomal endosomes: when ACAP2 is down-regulated by shRNA, dramatic reduction occurs in the neurite length [14]. In this scenario, truncated ACAP2 may disrupt primary neurulation, lead to nonclosure of neural tube, and finally cause myelomeningocele. Further investigations are required to clarify the exact function of *XXYTL1* and *ACAP2* during development, as well as their pathogenic roles.

Additionally, we compared the clinical features in our patient with previous cases diagnosed with CM-II and Sprengel’s deformity (Table 2) [12]. Although the genotypes were not available for all these cases, we found multiple segmentation defects of the spine, ribs, and shoulder among these patients, which suggests that these symptoms might result from a common genetic defect affecting somitogenesis.

**Table 2:** Clinical Findings in Patients with CM-II and Sprengel’s Deformity

Case	Gender	Clinical Observations	Age at surgical MMC/SsD correction
1	F	MMC, Left-SsD, Dextroconvex thoracic scoliosis, CM-II, Hypoplastic left thumb.	5 days/7 years
2	M	MMC, Right-SsD, Sinistroconvex thoracolumbar Scoliosis CM-II, Hydrocephalus Fused Th3–4-5 vertebral bodies	3 days/6 years
3	F	MMC, Right-SsD, Dextroconvex thoracolumbar scoliosis CM-II, Hydrocephalus Fused C7–Th1 vertebral bodies Fused right first to second ribs	2 days/ Not Applicable
This Case	F	MMC, Right-SsD, Mild thoracolumbar scoliosis, CM-II, No fused vertebral bodies or ribs observed,	2 years/7 years

*F* female, *M* male, *SsD* Sprengel’s deformity, *MMC* myelomeningocele, *CM-II* Chiari type II malformation

## Conclusion

The spinal defects in combination with Sprengel’s deformity occur very rarely, and thus far only few cases have been reported. What’s more, relevant developmental etiology and genetic causes still remain poorly understood. Although pathogenesis of both spinal dysraphism and Sprengel’s deformity remain unclear, there is evidence suggesting that both conditions might be linked to abnormal somitogenesis. In this case, deleted *XXYTL1* and *ACAP2* were identified in a patient with CM-II and Sprengel’s deformity. These two genes could have a critical role during somitogenesis, and their dysfunction may disrupt embryonic development, and lead to spinal dysraphism and shoulder defects. Further investigations are required to clarify their exact function during development, as well as their role in spinal dysraphism and Sprengel’s deformity.

## Abbreviations

CM-II: Chiari Malformation type II
CNV: Copy Number Variation
EGF repeats: Epidermal Growth Factor-like Repeats
GXYLT1/2: Glucoside Xylosyltransferase-1 or − 2
POGLUT-1: Protein O-glucosyltransferase 1
XXYTL1: Xyloside Xylosyltransferase 1
